# Salt tolerance mechanisms in the *Lycopersicon* clade and their trade-offs

**DOI:** 10.1093/aobpla/plab072

**Published:** 2021-12-08

**Authors:** Maria-Sole Bonarota, Dylan K Kosma, Felipe H Barrios-Masias

**Affiliations:** 1 Department of Agriculture, Veterinary and Rangeland Sciences, University of Nevada, Reno, NV 89557, USA; 2 Department of Biochemistry and Molecular Biology, University of Nevada, Reno, NV 89557, USA

**Keywords:** Crop improvement, salinity stress, tomato wild relatives

## Abstract

Salt stress impairs growth and yield in tomato, which is mostly cultivated in arid and semi-arid areas of the world. A number of wild tomato relatives (*Solanum pimpinellifolium*, *S. pennellii*, *S. cheesmaniae* and *S. peruvianum*) are endemic to arid coastal areas and able to withstand higher concentration of soil salt concentrations, making them a good genetic resource for breeding efforts aimed at improving salt tolerance and overall crop improvement. However, the complexity of salt stress response makes it difficult to introgress tolerance traits from wild relatives that could effectively increase tomato productivity under high soil salt concentrations. Under commercial production, biomass accumulation is key for high fruit yields, and salt tolerance management strategies should aim to maintain a favourable plant water and nutrient status. In this review, we first compare the effects of salt stress on the physiology of the domesticated tomato and its wild relatives. We then discuss physiological and energetic trade-offs for the different salt tolerance mechanisms found within the *Lycopersicon* clade, with a focus on the importance of root traits to sustain crop productivity.

## Teaser

Soil salinity affects ~20 % of cultivated land, and saline areas are expanding at a rate of ~10 % per year, compromising vegetable crop yields such as tomato (*Solanum lycopersicum*). Among the 12 tomato wild relatives, several species are a genetic resource for salt tolerance traits. In this review, we provide an update of the most recent literature about physiological traits that could be used in tomato salt tolerance breeding programs. We focus on root traits that could be key in promoting plant growth and yield under salt stress.

## Introduction

Salinity is one of the most challenging environmental conditions that limit crop growth and yield. It is estimated that more than 20 % of cultivated lands are affected by high-salinity conditions (Machado and Serralheiro 2017). Crop yield losses due to salinity amount to $26 billion per year (Singh et al. 2016). Soil salinization can occur via natural processes such as the rising of the water table, accumulation of salts on the soil surface as water evaporates, or by human activities (Shahid *et al.* 2018). As water availability for agriculture decreases or becomes erratic due to climate change, the use of poor-quality irrigation water, high rates of evapotranspiration and inappropriate use of fertilizers can exacerbate soil salinization, especially in arid and semi-arid regions. Saline areas are expanding at a rate of ~10 % per year (Foolad 2004; Liu *et al.* 2020). A soil is generally considered saline when the electrical conductivity (EC) of the saturation extract in the root zone exceeds 4 dS m^−1^ at 25 °C (Thorne, 1954). Saline soils can be managed with the use of high-quality water to leach the salts away from the root zone, but this method has become unsustainable and impractical (Boretti and Rosa 2019). Thus, the search for salt-tolerant traits compatible with crop productivity becomes necessary to reduce food insecurity under the increasingly water-limited and saline conditions of the future (IPCC 2019).

Tomato (*Solanum lycopersicum*) is the most important vegetable crop in the world, with its $119.6 billion production value in 2019 (FAOSTAT 2019), thanks to the versatility of its fruit, which can be consumed fresh, dried and processed, and to its nutritional and organoleptic characteristics. Global annual tomato production equates to roughly 243 million tonnes of fresh fruit produced on 6.1 million hectares in 166 countries (FAOSTAT 2019). Taxonomically, tomato belongs to the *Lycopersicon* clade, which includes 12 wild relatives (Knapp and Peralta 2016), that are all native to South America (Table 1). Members of this clade occupy a vast range of contrasting environments, ranging from sea level (e.g. Galapagos Islands) to ~3300 metres above sea level (e.g. Andean highlands), and from xeric to mesic environments (Taylor 1986). Although domesticated tomato is endemic to the same region, the domestication process has narrowed the vast genetic background to less than 5 % of that found in its wild relatives (Miller and Tanksley 1990). The domestication process typically results in larger edible organs (fruit or leaf), more vigorous plants, increased apical dominance, and decreased or loss of photoperiodicity and seed dormancy; however, the trade-off is usually an increased vulnerability to abiotic and biotic stresses (Yang *et al.* 2019). The process of tomato domestication has targeted numerous traits related to crop management and productivity, including growth habit (e.g. self-pruning gene for mechanical harvest), early flowering and concentrated fruit set, larger fruit size, fruit shape and colour, and crop disease resistance (Stevens and Rick 1986; Bai and Lindhout 2007; Labate *et al.* 2007; Barrios-Masias and Jackson 2014). Advance in breeding technologies such as markers, quantitative trait loci (QTL) mapping, genome-wide association studies (GWAS) and the use of introgression lines has enabled the widespread use of wild tomato species for breeding purposes (for an extensive review, see Labate *et al.* 2007 and Chaudhary *et al.* 2019). The utility of wild species for tomato breeding lies in the immense genetic and phenotypic variation, which has been largely investigated for abiotic and biotic stress tolerance traits (Table 2).

**Table 1.** Wild tomato relatives (*Solanum* section *Lycopersicon*) distribution across the Andean region. Data adapted from the Tomato Genetic Resource Center Database (https://tgrc.ucdavis.edu/).

Country	Tomato wild relatives
Chile	*S. chilense*, *S. peruvianum*
Ecuador	*S. habrochaites*, *S. neorickii*, *S. pimpinellifolium*
Ecuador (Galapagos Island)	*S. galapagense*, *S*. *cheesmaniae*, *S. pimpinellifolium*
Peru	*S. arcanum*, *S. chilense*, *S. chmielewskii*, *S. corneliomulleri*, *S. habrochaites*, *S. huaylasense*, *S. neorickii*, *S. pennellii*, *S. peruvianum*, *S. pimpinellifolium*

**Table 2.** Comparison of wild and domesticated tomatoes (*Solanum* section *Lycopersicon*) based on habitat and importance for breeding purposes. Data adapted from Peralta *et al.* (2005); Labate *et al.* (2007); Bergougnoux (2014); Grandillo *et al.* (2011).

Species		Habitat	Importance for tomato breeding	References
*S. lycopersicum*		Known only from cultivation or escapes; many escaped plants have smaller fruits (‘*cerasiforme*’); sea level to 4000 m	Moisture tolerance, resistance to wilt, root-rotting and leaf-spotting fungi	Hutton *et al.* (2010); Wang *et al.* (2013)
*S. pimpinellifolium*		Dry coastal habitats; 0–500 m, but exceptionally up to 1400 m	Colour and fruit quality; resistance to insect, nematode and diseases; drought and salt tolerance	Salinas *et al.* (2013); Villalta *et al.* (2008); Foolad (2007)
*S. chilense*		Hyper-arid rocky plains and coastal areas	Drought and salt tolerance; virus resistance	Kashyap *et al.* (2020); Tapia *et al.* (2016); Agrama and Scott (2006)
*S. pennellii*		Dry rocky hillsides and sandy areas; sea level to 2300 m	Drought and salt tolerance; resistance to insects	Gong *et al.* (2010); Frary *et al.* (2010)
*S. habrochaites*		On the western slopes of the Andes; in a variety of forest types, from premontane forest to dry forests; 200–3300 m	Cold and frost tolerance; resistance to insects due to their glandular hairs	Liu *et al.* (2012); Momotaz *et al.* (2010)
*S. cheesmaniae*		Arid, rocky slopes, prefers shaded sites; sea level to 1500 m	Salt tolerance; *Lepidoptera* and virus resistance	Villalta *et al.* (2008); Ali *et al.* (2019)
*S. galapagense*		Arid, rocky slopes, sometimes near shoreline within range of sea spray; sea level to 650 m	Salt tolerance; *Lepidoptera* and virus resistance	Firdaus *et al.* (2013); Pailles *et al.* (2020)
*S. neorickii*		Dry Andean valleys, moist, well-drained rocky slopes; 1950–2600 m	Resistance to *Botrytis cinerea* and *Oidium lycopersici*	Finkers *et al.* (2008); Faino *et al.* (2012)
*S. chmielewskii*		High dry Andean valleys; 1600–3200 m	Fruit quality	Ballester *et al.* (2016)
*S. peruvianum* north	*S. arcanum*	Coastal and inland Andean valleys; lomas formations, dry valleys and dry rocky slopes; 100–2800 m	Resistance to virus, bacteria, fungi, aphids and nematodes; salt stress tolerance	Anbinder *et al.* (2009); Tapia *et al.* (2016)
	*S. huaylasense*	Rocky slopes; 1700–3000 m		
*S. peruvianum* south	*S. peruvianum*	Coastal lomas formations and occasionally in coastal deserts, sometimes near agricultural fields as weed; sea level to 600 m		
	*S. corneliomulleri*	Rocky and sandy slopes; 200–3300 m		

Several tomato wild relatives such as *S. pimpinellifolium*, *S. chilense*, *S. pennellii, S. cheesmaniae* and *S. galapagense* are naturally adapted to highly saline soils. Under saline conditions, these species can show less decrease in growth, a greater capacity for achieving ion homeostasis in roots and shoots, a higher capacity to accumulate Na^+^ and Cl^−^ in leaves, enhanced hormonal signalling responses and osmotic adjustment, and more antioxidant activity when compared with cultivated tomato under salt stress (Frary *et al.* 2010; Albaladejo *et al.* 2017; Gharbi *et al.* 2017; Pailles *et al.* 2020). These characteristics make tomato wild relatives a potential genetic resource for improving salt tolerance in the cultivated tomato. However, the introgression of traits from wild relatives to elite cultivars remains challenging due to the complexity of salt tolerance traits, which can result in the transfer of loci deleterious to plant development and yield, making difficult to recover the genetic background of the elite cultivar in which the traits are being introgressed (Cuartero *et al.* 2006; Gharsallah *et al.* 2016; Dariva *et al.* 2020).

This review aims to compare salinity effects on physiological and biochemical aspects of the domesticated tomato and its wild relatives. We highlight the potential for wild relatives to contribute to improved salt tolerance of the cultivated tomato by discussing root and leaf traits associated with salt tolerance and their associated energetic trade-offs.

## Impact of Salinity Stress on Plant Growth and Physiology

Salt stress impairs not only growth and yield, but also many aspects of tomato plant physiology, such as plant water relations and metabolic processes. The impairment of tomato physiology under saline conditions differs depending on the developmental stage (Foolad 2007).

### Seed germination

Salt tolerance at the seed germination stage depends on the seed ability to withstand the effects of high concentrations of soluble salts and lower soil water potential (Bradford, 1995). The seed must generate sufficient osmotic potential to improve the water status of the embryo (i.e. imbibition stage), and allow the biochemical processes that enable radicle growth and germination. In tomato, high salinity delays the onset and reduces the rate and final percentage of germination (Singh *et al.* 2012; Tahir *et al.* 2018). The germination rate of tomato cultivars is significantly impaired at 100 mM NaCl (~10 dS m^−1^) (Tahir *et al.* 2018). A number of wild tomato relatives exhibit greater salt tolerance compared to the cultivated tomato at the germination stage (Jones 1986a; Foolad and Jones, 1991; Foolad and Lin 1997; Cuartero and Fernandez-Munoz 1999). For instance, *S. pimpinellifolium* (accession LA722), *S. peruvianum* (accession PI127832) and *S. pennellii* (accession LA716) showed higher germination rates compared with cultivated tomato at 100 mM NaCl (~10 dS m^−1^) (Jones 1986a). The tomato seed embryo is coated by two protective tissues (endosperm and the testa), which must be at least partially degraded for the completion of germination. This degradation is catalysed by hydrolytic enzymes such as β-mannosidase and α-galactosidase, which are known to be inhibited under salt stress (Ali and Elozeiri 2017). A study of the tomato cv. ‘Ciettaicale’ and cv. ‘San Marzano’ revealed that ‘Ciettaicale’ seeds maintained higher endo-β-mannanase, β-mannosidase and α-galactosidase activities under mild salt stress conditions (25 mM NaCl or ~2.5 dS m^−1^). These elevated glycosyl hydrolase activities were accompanied by higher antioxidant activity as well as greater starch mobilization and higher total soluble sugar accumulation. These changes were especially prominent in ‘Ciettaicale’ at 72 h post salt exposure. Overall, ‘Ciettaicale’ exhibited a higher percentage and rates of germination compared with ‘San Marzano’ (Moles *et al.* 2019).

### Vegetative stage

**Figure 1.**
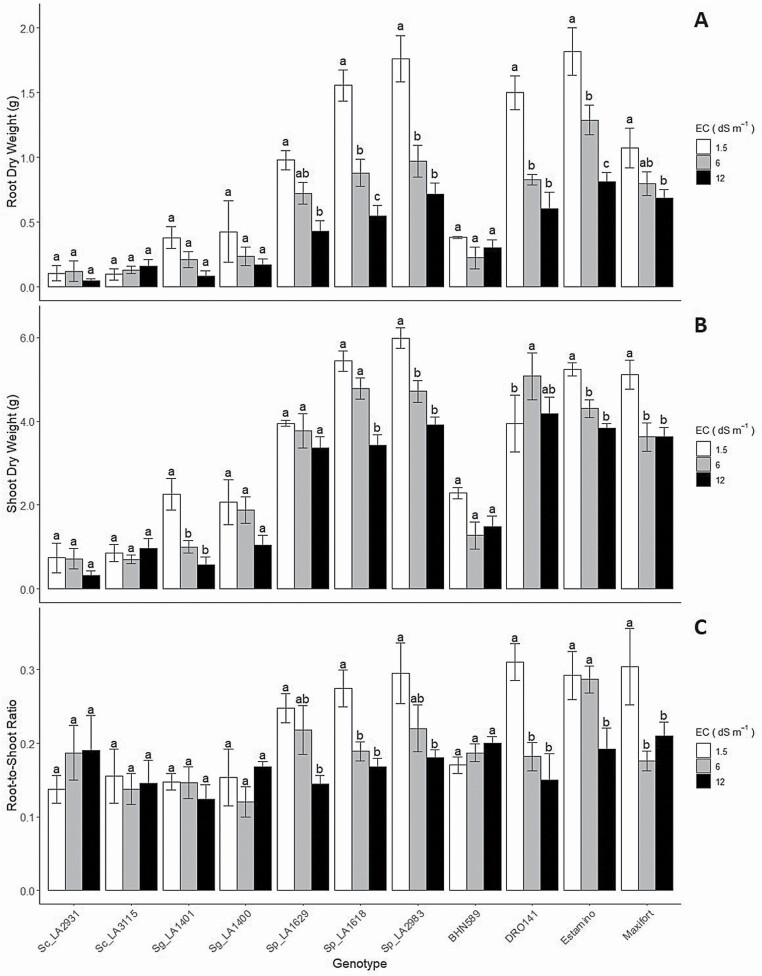
Root (A) and shoot (B) dry weight and root-to-shoot ratio (C) from a salt tolerance screening of the tomato wild relatives *S. chilense* (accessions LA2931, LA3115), *S. galapagense* (accessions LA1400, LA1401) and *S. pimpinellifolium* (accessions LA1618, LA1629, LA2983), a commercial tomato cultivar (‘BHN589’) and three commercial rootstocks (DRO-141TX, Estamino, Maxifort). Three levels of salinity were applied (0, 60 and 120 mM NaCl, corresponding to ~1.5, 6 and 12 dS m^−1^). Data were analysed with two-way ANOVA using anova function in R 3.6.3 (R Core Team 2021). Data were transformed as necessary when ANOVA assumptions were not met. For all models, the alpha for the main effect was set at 0.05 level. When the calculated *P*-value was lower than our chosen alpha, the null hypothesis was rejected and a *post hoc* multiple-comparison procedure was conducted with the multcomp function (emmeans package) using the Bonferroni method. Mean comparisons are within genotype. Values are mean ± standard error (*n* = 3–9).

During the vegetative stage, shoot and root growth are impaired under salt stress due to restrictions in cell expansion, which are the result of low soil water potential, nutrient imbalance and ion toxicity. For example, at an early vegetative stage (3–4 leaves stage) *S. lycopersicum* cv. ‘Heinz 1350’ and ‘VF234’ exhibit 60 % reduction in stem elongation rate under saline conditions. By contrast, wild tomato relatives grown under the same conditions exhibited smaller reductions in stem elongation (e.g. *S. pennellii* accession Atico: 55 %, and *S. cheesmaniae* ecotype no. 1400: 32 %) (Tal and Shannon 1983). One characteristic typical of salt-tolerant tomato wild relatives is that they show less growth under non-saline conditions, but they outperform the domesticated tomato under high levels of salt stress. For instance, at 3–4 leaves stage, the tomato cv. ‘Moneymaker’ showed a greater shoot biomass and growth rate than *S. pennellii* accession PE-47 during the first 7 days of salt treatment (100 mM NaCl, or ~10 dS m^−1^). However, after 7 days *S. pennellii* accession PE-47 outperformed the commercial cultivar (Albaladejo *et al.* 2017). At the 7–8 leaf stage, a comparison between 28 accessions of *S. galapagense*, 39 of *S. cheesmaniae* and two tomato cultivars (Heinz 1706 and Moneymaker) revealed that all wild relative accessions had a lower decrease in total plant biomass under salt stress compared with its control when exposed to saline conditions for a 10-day period in a hydroponic system (200 mM NaCl, or ~20 dS m^−1^; Pailles *et al.* 2020). In our evaluations of a commercial tomato cultivar (cv. ‘BHN589’), three commercial rootstocks (DRO-141TX, Estamino, Maxifort) and the wild relatives *S. chilense* (accessions LA2931, LA3115), *S. galapagense* (accessions LA1400, LA1401) and *S. pimpinellifolium* (accessions LA1618, LA1629, LA2983) exposed to moderate (6 dS m^−1^) and severe (12 dS m^−1^) salinity at the 5–6 leaf stage revealed wide differences in biomass production and carbon allocation after 3 weeks of salinity treatment (Fig. 1). Two accessions each of *S. chilense* and *S. galapagense* showed significantly less root and shoot biomass accumulation compared to the cultivated tomato (cv. ‘BHN589’), the three commercial tomato rootstocks and the four accessions of *S. pimpinellifolium*, under both control and salt stress conditions. *Solanum pimpinellifolium* is the closest wild relative to the domesticated tomato (Peralta and Spooner 2005), which could explain their more similar behaviour in biomass accumulation under salt stress as compared to *S. galapagense* and *S. chilense*. The tomato rootstocks exhibited higher shoot and root biomass than the cv. ‘BHN589’ under salt stress, suggesting that grafting could be an effective technique to increase tolerance to several abiotic stresses (Singh *et al.*, 2020; Bristow *et al.* 2021). *Solanum pimpinellifolium* showed higher shoot and root biomass than the cv. ‘BHN589’, but similar to the commercial rootstocks. The relative decrease in root and shoot growth, compared to the control (1.5 dS m^−1^), was lower in the wild relatives *S. chilense* and *S. galapagense* than in tomato rootstocks and *S. pimpinellifolium*. The root-to-shoot ratio, which is usually related to salt tolerance in the *Lycopersicon* clade (Taleisnik 1987; Cruz and Cuartero 1990; Maggio *et al.* 2007), was maintained under salt stress in *S. chilense*, *S. galapagense* and the cv. ‘BHN589’, but decreased in the tomato rootstocks and *S. pimpinellifolium*.

### Reproductive stage

Whereas mild salt stress has been demonstrated to improve fruit quality (Meza *et al.* 2020), high soil salinity is responsible for decreases in tomato crop yields (De Pascale *et al.* 2003; Massaretto *et al.* 2018). Flower development under salt stress seems to be more impaired by a disequilibrium between source and sink organs, due to the accumulation of carbohydrates in the leaves instead of ion toxicity in reproductive organs (Balibrea *et al.* 2003; Ghanem *et al.* 2009). In tomato, fruit size is more affected than fruit number under moderate salt concentrations, but both decrease under severe salt stress (Cuartero and Fernandez-Munoz 1999). A study comparing 94 accession of *S. pimpinellifolium* and two tomato cultivars (cv. ‘Arka Meghali’ and the inbred line CA4) showed that the *S. pimpinellifolium* had a larger decrease in fruit number than fruit weight, resulting in a lower per plant yield reduction (~82 %) compared to the two tomato cultivars (>87 % decrease) (Rao *et al.* 2013). A comparison of *S. lycopersicum* var. *cerasiforme* and *S. chilense* (accession LA4107) revealed that *S. chilense* had less of a decrease in fruit size than the domesticated tomato (Martinez *et al.* 2012). Increasing the capacity of the plant to maintain a lower fruit osmotic potential (Ψ _*s*_) could allow fruit growth under salt stress (Bolarin *et al.* 2001). A comparison between the tomato cv. ‘Moneymaker’ and *S. pimpinellifolium* (accession PE-2) showed that the latter was able to use Na^+^ and Cl^−^ to decrease fruit Ψ _*s*_ and maintain fruit development under salinity conditions, whereas the domesticated tomato used sugars for osmotic adjustment (Bolarin *et al.* 2001), with higher carbon and energy costs (Tyerman *et al.* 2019).

## Salt Tolerance Mechanisms and Their Trade-offs

Energy demands under salt stress increase due to elevated photorespiration, protein turnover and ion transport activity (Wingler *et al.* 2000; Miller *et al.* 2010; Shi and Theg 2013). The ability of a plant to maintain growth and survive under salt stress is considered salinity tolerance, and two main mechanisms for salt tolerance in the *Lycopersicon* clade have been identified: (i) salt extrusion, which prevents Na^+^ movement to the shoot through active exclusion from or retention in the roots; and (ii) salt inclusion, where tissue tolerance is achieved through compartmentalization in vacuoles to avoid toxicity, and accumulation of compatible osmolytes to balance the osmotic pressure in the cytosol (Shabala 2013; Almeida *et al.* 2014). Whereas *S. lycopersicum* generally employs an extrusion mechanism to cope with soil salinity, several wild accessions adapted to saline areas, from wild relatives such as *S. pimpinellifolium*, *S. cheesmaniae*, *S. galapagense*, *S. pennellii* and *S. peruvianum* generally utilize an inclusion mechanism (Foolad 2004). However, different reports show discrepancies about this distinction, which can result from the use of different accessions, using plants at different developmental stages and different forms of salinity treatments (Rajasekaran *et al.* 2000). For this reason, we will consider the distinction between exclusion and inclusion mechanisms as not specific to a tomato cultivar or wild relative species. Different salt tolerance mechanisms have different energetic costs for the plant, which can affect the overall plant growth and yield. The capacity of the plant to efficiently use its energy resources is essential to ensure higher growth and yield under salinity conditions. The maintenance of a favourable plant water status and nutrient uptake rate are also fundamental for maintaining growth and yield. Overall, the concentration of Na^+^ in tomato shoots is not a good indicator of performance under salt stress (Saranga *et al.* 1991; Almeida *et al.* 2014), but a suite of biochemical, physiological and anatomical traits can be involved in conferring salt tolerance. Several root and leaf traits that are key for maintaining nutrient balance, plant water status, an efficient use of energy resources and preventing ion toxicity under salt stress are discussed herein and summarized in Table 3.

**Table 3.** Phenotypes and associated genes or proteins found in the domesticated tomato and its wild relatives for salt tolerance breeding purposes.

Phenotype	Associated genes or proteins	Tomato species	Effect in salt tolerance	References
Nitrogen uptake and assimilation	*AMT1.1*, *AMT1.2*, *GS1*	*S. pennellii*	Smaller reduction in biomass	Abouelsaad *et al.* (2017)
Root hydraulic conductivity	*TPX1* *PIP2;1*	*S. lycopersicum; S. pimpinellifolium*	Less incidence of hydraulic failure; better plant water status	Lucena *et al.* (2003); Han *et al.* (2020)
Primary root development	?	*S. lycopersicum; S. chilense; S. huaylense; S. galapagense*	Exploration of deeper layers of the soil, which are less saline	Alaguero-Cordovilla *et al.* (2018)
Lateral roots development	?	*S. pimpinellifolium*	Better access to less mobile nutrients, such as K^+^	Alaguero-Cordovilla *et al.* (2018)
Root apoplastic barriers	?	*S. lycopersicum*	Sodium interception and exclusion	Li *et al.* (2018)
Sodium extrusion to the apoplast	SOS pathway	*S. pimpinellifolium*	Less Na^+^ transport to the shoot	Sun *et al.* (2010)
Sodium partition	*HKT2;1*	*S. cheesmaniae*	Sodium xylem retrieval and phloem redistribution	Jaime-Perez *et al*. (2017)
Sodium/calcium ratio	*SlCLB10*	*S. lycopersicum*	Higher fruit production Less blossom end rot incidence	Egea *et al.* (2018)
Sodium/potassium ratio	*HAK*/*KUP*/*KT* *HKT1;2*	*S. lycopersicum; S. cheesmaniae*	Less Na^+^ transport to the shoot Less decrease in shoot growth	Wang *et al.* (2020); Romero-Aranda *et al.* (2020)
Sodium compartmentation	NHX antiporters	*S. chilense; S. pimpinellifolium*	Higher plant growth rate after long salt stress exposure	Kashyap *et al.* (2020); Gálvez *et al.* (2012)
Intracellular vesicular trafficking	RabGAP SNARE	*S. chilense; S. pimpinellifolium; S. pennellii*	Increased endocytosis and vacuolar Na^+^ compartmentalization	San Martin-Davison *et al.* (2017); Madrid-Espinoza *et al.* (2019)
Stomatal conductance Stomatal density	?	*S. pennellii*	Better water status of the leaf	Albaladejo *et al.* (2017)
Carbon metabolism	Sucrose synthase UTP-glucose-1-P	*S. chilense*	Higher photosynthetic rate and growth	Balibrea *et al.* (2003); Zhou *et al.* (2011)
Glycolysis	TPI, enolase, NAD-dependent glyceraldehyde-3-P	*S. chilense*	Reduced glucose level	Zhou *et al.* (2011)
Biosynthesis of BCAAs	Ketol-acid reductoisomerase	*S. chilense*	Energy regeneration, peptide elongation, glutamate recycling, fatty acid synthesis	Zhou *et al.* (2011)
Sodium contribution to osmotic adjustment	?	*S. chilense*	Higher efficiency in osmotic adjustment	Gharbi *et al.* (2017)
Organic osmolytes in osmotic adjustment	*P5CS*	*S. lycopersicum*	Higher proline accumulation in the leaves	Almeida *et al.* (2014)
Enzymatic antioxidant activity	SOD, CAT, APX, POX	*S. pennellii*	ROS scavenging	Frary *et al.* (2010)
Non-enzymatic antioxidant activity	Water-soluble antioxidant activity; phenols; flavonoids	*S. pennellii*	ROS scavenging	Frary *et al.* (2010)

### Effect of soil salinity

In soil, salinity results in particle disaggregation, clay dispersion and increased soil pH, initially leading to an impairment of nutrient availability for plants. Cultivated tomato is moderately sensitive to saline conditions and tolerates soils with an EC of 2.5 dS m^−1^ (~25 mM NaCl). Above this threshold, crop productivity is reduced at a rate of ~10 % per unit increase of EC (~10 mM NaCl increase) (Saranga *et al.* 1991). Under salt stress, tomato growth is initially impaired by nutrient imbalance and osmotic effects, and later by the toxicity of high intracellular ion concentrations (especially Na^+^) (Cuartero and Fernandes-Munoz 1999). Moderately high concentrations of Na^+^ and Cl^−^ (EC = 3–5 dS m^−1^) in the soil solution inhibit the uptake of ions such as NH_4_^+^, NO_3_^−^, K^+^ and Ca^2+^ (Munns 2002; Aktas *et al.* 2006). The inhibition of N uptake might occur by antagonism between NH_4_^+^/Na^+^ and NO_3_^−^/Cl^−^ (Shawer 2014), reduction in plant water uptake capacity, depolarization of cell membranes by high Na^+^ concentration (Suhayda *et al.* 1990) and downregulation of genes responsible for NH_4_^+^ assimilation (Wang *et al.* 2012). A comparison between the domesticated tomato (cv. ‘Manitoba’) and its wild relative *S. pennellii* (accession LA0716) showed that salt stress inhibited the uptake and assimilation of nitrate in both species; however, *S. pennellii* presented a smaller reduction in biomass and higher survival rate than cv. ‘Manitoba’, which was attributed to the higher expression of key genes involved in ammonium uptake (*AMT1.1* and *AMT1.2*) and assimilation (*GS1*) (Abouelsaad *et al.* 2016). The strong membrane depolarization induced by high Na^+^ concentration (60–80 mV for 100 mM NaCl) leads to increased efflux and reduced influx of K^+^ (Shabala and Cuin 2008). As K^+^ has important structural, signalling and metabolic functions in plant physiology, a high Na^+^/K^+^ ratio results in nutritional imbalance (Cramer 2002; Tuna *et al.* 2007). Potassium is involved in osmotic adjustment, enzyme activation, photosynthesis and respiration, and stomatal regulation (Maach *et al.* 2020). As such, maintaining a low Na^+^/K^+^ cytosolic ratio in the shoot can result in higher salt tolerance in tomato. The addition of 2 mM K^+^ to high salt nutrient solutions (100 and 200 mM NaCl; ~10 and 20 dS m^−1^) was shown to decrease Na^+^ accumulation in the shoot and diminish salt-induced growth inhibition in the domesticated tomato (Al-Karaki 2008). This is likely due to the competition between Na^+^ and K^+^ for translocation to the shoot (Shabala and Cuin 2008). Tomato wild relatives, such as *S. pennellii*, *S. chilense*, *S. galapagense* and *S. cheesmaniae*, have been studied in relation to ion selection and transport and their higher capacity to withstand high Na^+^/K^+^ ratios in the leaves (Bolarin *et al.* 1995; Gharbi *et al.* 2017; Jaime-Perez *et al*. 2017; Pailles *et al.* 2020). The maintenance of a low cytosolic Na^+^/Ca^2+^ ratio is also important under salt stress. For instance, the tomato *SlCLB10* (Calcineurin b-like protein 10, important for balance the Na^+^/Ca^2+^ ratio) knock-out mutants showed impaired plant growth and fruit production as well as increased blossom end rot incidence under salt stress (Egea *et al.* 2018).

### Root water uptake

The root system, being the first organ to be exposed to salt stress, has a critical function in sustaining canopy water demands needed for carbon assimilation and plant growth, and plays a key role in crop salt tolerance in horticultural systems (Barrios-Masias *et al.* 2019). In cultivated tomato, the root apoplastic water flow is 12-fold larger than the cell-to-cell water flow under well-watered conditions (Hernandez-Espinoza and Barrios-Masias 2020). Under salt stress, however, transpiration is limited and the tension that drives water through the apoplastic pathway is reduced. The overall root hydraulic conductivity (*Lp*_r_) decreases linearly with increasing soil salinity in tomato plants (Rodriguez *et al.* 1997). Lower *Lp*_r_ in tomato was related to the overexpression of a basic peroxidase gene (*TPX1*), whose transcripts are primarily expressed in the epidermal and subepidermal cells of mature roots, and whose expression is also induced by >50 mM NaCl (~5 dS m^−1^) (Botella *et al.* 1994). Transgenic tomato overexpressing *TPX1* had lower total xylem area, higher ligno-suberization of root exodermis and lower root *Lp*_r_ (Lucena *et al.* 2003). Aquaporins also play a key role in tomato *Lp*_r_ and in tomato salt tolerance (Bramley *et al.*, 2009; Gambetta *et al.*, 2017; Jia *et al.* 2020). Aquaporin genes of the plasma membrane intrinsic protein (PIP) family have been identified in *S. lycopersicum* and their induction has been shown to improve leaf water status (Li *et al.* 2016). Under salt stress, the expression of *PIP2;1* was significantly lower in the roots of *S. pennellii* (accession PE-47) compared to the tomato cv. ‘Moneymaker’ after 2 days of salt treatment, but significantly higher in the leaves, allowing for a better regulation of water use at the root level, and better leaf water status under salt stress (Albaladejo *et al.* 2017). A comparison between *S. pimpinellifolium* (accession L03708) and the tomato cv. ‘M82’ showed that the former had a higher capacity to maintain *Lp*_r_ under salt stress which was attributed, in part, to *S. pimpellifolium*’s capacity to maintain root *PIP1* transcript abundance (150 mM NaCl, ~15 dS m^−1^) (Han *et al.* 2021). Root *Lp* is a complex trait, and variability within the *Lycopersicon* clade has been found, which warrants further study to provide a more mechanistic understanding of salt tolerance in tomato.

### Root system architecture

Soil salts concentrate on the soil surface as a result of soil water evaporation. Root system architecture (RSA) is another important trait that confers plasticity in soil exploration and the regulation of water and nutrient uptake, and can enhance plant salt tolerance (Jung and McCouch 2013). Root system architecture refers to the magnitude and pattern of root branching and growth, internode length, and root angles and diameter (Fitter 1991). Genetic and metabolic pathways involved in RSA modulation have been studied primarily in *Arabidopsis* and cereals (Jung and McCouch 2013; Rogers and Benfey 2015), but less so in vegetable crops. Many factors are involved in modulating RSA, such as hormones, their receptors and transcription factors that respond to environmental signals (reviewed in Jung and McCouch 2013). For instance, the gene *DEEPER ROOTING 1* (*DRO1*) is associated with the regulation of root deepening and root angles in *Oryza sativa* (Uga *et al*. 2011, 2013), and *DRO1* orthologs have been identified in *Prunus* species, *Arabidopsis* and wheat (Guseman *et al.* 2017). In domesticated tomato, exposure to salt stress induced a reduction in root length and weight, but led to an increase in the number of fine roots (Lovelli *et al.* 2012), suggesting that RSA is involved in tomato response to salt stress. Variability in RSA between tomato and its wild relatives during early growth stages has been demonstrated under non-saline conditions (Alaguero-Cordovilla *et al.* 2018). Two tomato cultivars (cv. ‘Ailsa Craig’, and cv. ‘Moneymaker’) and the wild relatives *S. chilense* (LA1932), *S. huaylense* (LA2663), *S. galapagense* (LA1044) showed higher primary root elongation rate, whereas the tomato cv. ‘Craigella’, the wild relatives *S. pimpinellifolium* (LA1587), *S. corneliomulleri* (LA1274), *S. peruvianum* (LA1336), *S. cheesmaniae* (LA1037) and *S. arcanum* (LA2157) exhibited intermediate primary root growth rate, and *S. chmielewskii* (LA2663) had a lower root elongation rate. Interestingly, the domesticated tomato cultivars showed a higher lateral root number and growth rate after root-tip excision compared to most of the wild accessions; except for *S. pimpinellifolium* and *S. peruvianum*, which had higher and similar lateral root numbers, respectively. The angle of lateral roots was significantly higher in *S. arcanum* and tomato cv. ‘Moneymaker’. This phenotypic response may be related to genetic variability in the *DRO1* pathway (Alaguero-Cordovilla *et al.* 2018). Root system architecture variability within the *Lycopersicon* clade should be further investigated in relation to salinity tolerance as soil salinity changes across the soil profile.

### Passive exclusion of Na^+^ from the root stele

Root apoplastic barriers (e.g. Casparian strip and aliphatic suberin) are thought to be involved in salt homeostasis at the root level by favouring water uptake through the cell-to-cell pathway (Steudle 2000; Blum 2011; Knipfer and Fricke 2011; Barrios-Masias *et al.* 2015), resulting in higher ion selectivity. In *Arabidopsis*, several genes involved in Casparian strip development have been identified (Kamiya *et al.* 2015). Furthermore, knocking out the gene *CYP86A1/HORST*, which encodes a cytochrome P450-dependent fatty acid ω-hydroxylase, resulted in 60 % decrease in aliphatic suberin, increased salt sensitivity and Na^+^ accumulation in the shoots and the roots compared to the wild-type *Arabidopsis* under salt stress (15 and 30 mM NaCl after 3, 7 and 14 days) (Wang *et al.* 2020). Li *et al.* (2018) studied the regulatory pathways for tomato Casparian strips, and they concluded that there is a functional conservation in the regulatory mechanism of Casparian strips formation between *A. thaliana* and the cultivated tomato. However, tomato has a more complex root structure (e.g. presence of exodermis), and to understand the role of root apoplastic barriers, as they develop closer to the root tip in response to salinity, more research is required.

### Active exclusion of Na^+^ from the shoot

At the root level, Na^+^ extrusion is regulated by a group of Na^+^/H^+^ antiporters encoded by the *SOS* gene family, which can both extrude Na^+^ to the soil and regulate the Na^+^ partition between different plant organs (Munns and Tester 2008; Olías *et al.* 2009). The signal transduction pathway of the SOS proteins is based on cytosolic Ca^2+^ concentrations. The increase of Ca^2+^ in the cytosol elicited by salinity is sensed by SOS3, which interacts with SOS2 and activates the plasma membrane Na^+^/H^+^ exchanger encoded by the *SOS1* gene, operating to re-establish Na^+^ homeostasis in cells through extrusion of Na^+^ into the apoplast or xylem vessels (Ishitani *et al.* 2000; Olías *et al.* 2009). Sodium extrusion is an energetically demanding process. For each mole of Na^+^ effluxed from the cell, the hydrolysis of one mole of ATP is required to power the movement of an H^+^ into the cell by the plasma membrane H^+^-ATPase in order to maintain membrane potential (Malagoli *et al.* 2008; Munns *et al.* 2020). Sun *et al.* (2010) demonstrated that *S. pimpinellifolium* (accession PI365967) has a more active SOS pathway and lower accumulation of Na^+^ in the shoot relative to Na^+^ in the root, compared to *S. lycopersicum* cv. ‘Moneymaker’, suggesting that the SOS pathway plays a role in intercepting Na^+^ translocation to the shoot. However, in a study comparing 23 accessions of tomato wild relatives and commercial cultivars, the *SOS1* expression in the roots did not correlate with Na^+^ accumulation in leaves, stems or roots (Almeida *et al.* 2014). In tomato, Na^+^ homeostasis and partitioning is also regulated by *HKT1*-like transporters (Asins *et al.* 2013). These xylem parenchyma, plasma membrane-localized transporters function to retrieve Na^+^ from xylem vessels to the xylem parenchyma cells thereby preventing xylem-mediated transport of Na^+^ to the leaves (Sunarpi *et al.* 2005). Two near-isogenic lines (NIL) homozygous for the *S. chilense* and *S. lycopersicum* var*. cerasiforme* alleles at both *HKT1* loci (*HKT1;1* or *HKT1;2*) were used to create transgenic lines silenced in either *HKT1;1* or *HKT1;2* genes, resulting in salt hypersensitivity (Jaime-Perez *et al*. 2017). However, the *HKT1;2* gene was shown to be more effective in controlling shoot Na^+^/K^+^ homeostasis compared to *HKT1;1* (Romero-Aranda *et al.* 2020). The silencing of the *HKT1;2* allele from *S. cheesmaniae* compared to the allele from *S. lycopersicum* showed a decrease in shoot growth, suggesting a better effectiveness of the former in salt tolerance (Jaime-Perez *et al*. 2017). The HAK/KUP/KT (high-affinity K^+^/K^+^ uptake/K^+^ transporter) family transporters are involved in both Na^+^ and K^+^ xylem loading in roots. Recently, a *SlHAK20* gene variant, which arose during tomato domestication, was associated with a decrease in Na^+^ versus K^+^ affinity resulting in less root-to-shoot movement of Na^+^ (Wang *et al.* 2020). Once Na^+^ is retrieved from the xylem sap, it can be compartmentalized into root cell vacuoles, redistributed towards sink organs and tissues through the phloem or extruded to the apoplast (Jaime-Perez *et al*. 2017); although the fate of the removed Na^+^ is not clear.

### Sodium vacuolar compartmentalization

Sodium vacuolar sequestration happens through the NHX antiporter family, which are (Na^+^, K^+^)/H^+^ antiporters that are involved in Na^+^ compartmentalization inside the vacuole, increased K^+^ retention in the cells, and osmotic adjustment by Na^+^ or K^+^ (Rodríguez-Rosales *et al.* 2008). An evaluation of 23 wild and cultivated tomato accessions for salinity tolerance showed that higher expression of *NHX1* and *NHX3* genes in roots experiencing salt stress was positively correlated with Na^+^ accumulation in roots and negatively correlated with Na^+^ accumulation in the shoots (Almeida *et al.* 2014). Concordantly, when compared with *S. lycopersicum*, *S. pennellii* (accession PE-47) plants experiencing salt stress had higher *NHX3* and *NHX4* expression levels in leaves and lower *NHX3* and *NHX4* transcript accumulation in the roots, and Na^+^ accumulation was lower in roots and higher in leaves (Albaladejo *et al.* 2017). In a transcriptome analysis of *S. chilense* (accession LA1972), *NHX1* gene was overexpressed in the shoot under severe salt stress (500 mM NaCl, EC 26.8 dS m^−1^) (Kashyap *et al.* 2020). Four NHX isoforms were studied in *S. pimpinellifolium* and the tomato cv. ‘Volgogradskij’ under salt stress (130 mM NaCl; ~13 dS m^−1^) (Gálvez *et al.* 2012). The former showed higher expression of the isoform *NHX1* in the leaves and the shoot, especially after 1 day of treatment, while cv. ‘Volgogradkij’ increased *NHX1* expression only in the leaves after 1 day of salt exposure. The isoforms *NHX2* and *NHX3* showed a similar pattern of expression and their expression increased more in *S. pimpinellifolium* (roots, leaves and stem) than in cv. ‘Volgogradskij’, especially after 7 days of salt exposure. The isoform *NHX4* was overexpressed especially in stems and fruits in both species, indicating its specific role for reproductive tissue. *Solanum pimpinellifolium* had higher accumulation of Na^+^ and lower K^+^ in the shoot compared to the tomato cv. ‘Volgogradskij’, and was able to maintain growth after 7 days of salt exposure (Gálvez *et al.* 2012).

### Intracellular vesicular trafficking

Under salt stress, lipids and proteins are mobilized between different organelles (Okazaki and Saito 2014), ion channels and transporters are removed from the plasma membranes (Yang and Guo 2018), reactive oxygen species (ROS) are detoxified (You and Chan 2015) and toxic ions are compartmentalized (Baral *et al.* 2015). For these functions, an efficient intracellular vesicular trafficking system is necessary, in both leaves and roots. An important family of enzymes involved in vesicular trafficking are the RabGTPases. They switch from the active state (bound to GTP) to inactive state (bound to GDP) (Rehman and Di Sansebastiano 2014). When they are active, they are recognized by effector proteins and facilitate membrane fusion (Vukašinović and Žárský, 2016). The introgression of the salt-induced gene *SchRabGDI1*, encoding for a protein regulator of the RabGTPase cycle, from *S. chilense* to *A. thaliana* conferred salt tolerance, and increased endocytosis and vacuolar Na^+^ accumulation compared to the wild-type *A. thaliana* (San Martin-Davison *et al.* 2017). The differential expression of the *RabGAP* genes (encoding for GTPase-activating proteins) in roots and shoots of *S. lycopersicum*, *S. pimpinellifolium* and *S. pennellii* under salt stress suggest a possible role for this gene in determining tomato salt tolerance (Madrid-Espinoza *et al.* 2019). Although the role of each RabGAP protein under salt stress is not clear, comparisons with the homologues from *A. thaliana* and *O. sativa* revealed that RabGAP21 could be localized in the Trans-Golgi network and the pre-vacuolar compartments, suggesting that it could have an important role in mobilizing transporters and proteins associated with the vacuole (Madrid-Espinoza *et al.* 2019). Soluble *N*-ethylmaleimide-sensitive factor attachment protein receptors (SNARE) are involved in membrane fusion and vesicular trafficking in plants. The expression of a number of *SNARE* genes in roots and leaves in response to salt stress has been analysed by qRT-PCR in *S. lycopersicum*, *S. pimpinellifolium* and *S. chilense*. This analysis revealed that SNARE proteins *SchSYP51.2*, *SchVAMP727* and *SchGOS12.2* transcript abundance increased more than 40 times after 12 h of salt exposure in the roots of the salt-tolerant *S. chilense* in contrast to *S. lycopersicum* and *S. pimpinellifolium*, which had only 1- to 8-fold increase (Salinas-Cornejo *et al.* 2019), suggesting that *S. chilense* may have a more active root vesicular trafficking system.

### Gas exchange regulation

Due to impaired water uptake capacity, transpiration under salt stress is inhibited, resulting in a lower C assimilation capacity. The maintenance of stomatal conductance during salt stress has been related to salt tolerance (Sanchez-Blanco *et al*. 1991). A study on 15 tomato cultivars showed that decreases in leaf gas exchange resulted in reduced shoot growth in all cultivars (Amjad *et al.* 2014). A comparison between the tomato cv. ‘M82’ and *S. pennellii* (accession PE-47) showed that the latter had higher leaf temperature increase, indicating lower transpiration, together with higher water content of the leaf after 7 and 14 days of salt exposure (100 mM NaCl, ~10 dS m-1) (Albaladejo *et al.* 2017). *Solanum pennellii* (accession PE-2) showed a significant decrease in abaxial and adaxial stomatal density under salt stress (Albaladejo *et al.* 2017), which can lead to lower transpiration rates in tomato (Farber *et al.* 2016). The trade-off between water saving and carbon assimilation (i.e. water use efficiency) may be counterproductive for biomass accumulation and yield (Khavari-Nejad and Mostofi, 1998), and should be further investigated in tomato and its wild relatives under salt stress. In addition to reductions in stomatal conductance, changes in metabolic processes involved in carbon fixation have been investigated under salt stress within the *Lycopersicon* clade. A proteomics analysis of the wild tomato relative *S. chilense* (accessions LA2747 and LA1958) showed a lower abundance of several Calvin cycle (i.e. sedoheptulose-1,7-bisphosphatase) and photorespiration enzymes (i.e. phosphoglycolate phosphatase, hydroxypyruvate reductase and glycolate oxidase) in the leaves of salt-treated plants (Zhou *et al.* 2011). The authors argued that reductions in the abundance of these enzymes could lead to a decrease in both carbon fixation and photorespiration to compensate for the low photosynthetic rate of *S. chilense* under salt stress, resulting in a higher energy use efficiency. Carbon metabolism and partitioning are impaired under saline conditions. A comparison between a salt-tolerant tomato ecotype (cv. ‘Pera’) and a salt-sensitive tomato (cv. ‘Volgogradskij’) showed that a higher accumulation of photoassimilates (fructose, glucose and sucrose) in the leaves was correlated with lower photosynthetic activity and growth in the salt-sensitive cultivar (Balibrea *et al.* 2000). Under salt stress, higher sink strength and cytoplasmatic sucrolytic activity resulted in higher sucrose metabolism and phloem unloading (i.e. assimilate transport to sink organs) in *S. cheesmaniae* (accession LA530) and *S. chmiliewskii* (accession LA1028) compared to the domesticated tomato cv. ‘H-324-1’ (Balibrea *et al.* 2003).

### Osmotic adjustment

Leaf osmotic adjustment is necessary for the maintenance of cell and organ turgor in plants experiencing salt stress. The relative contribution of inorganic ions versus compatible osmolytes to the overall osmotic adjustment will determine the energy costs involved in this tolerance mechanism. Under non-limiting conditions (e.g. no photorespiration), 3 ATP and 2 NADPH are utilized for each molecule of CO_2_ assimilated (Wingler *et al.* 2000). Under salt stress, the energetic requirements for CO_2_ assimilation increase to 5.375 ATP and 3.5 NADPH per molecule of CO_2_ fixed (Munns *et al.* 2020). Thus, the use of compatible osmolytes (e.g. organic solutes) to maintain high osmotic potential of the cytosol has considerable carbon and energy costs for the plant. Instead, the accumulation of inorganic ions is less energy demanding (Munns *et al.* 2020). Sodium contribution to osmotic adjustment in *S. chilense* (accession LA4107) was found to be more than 60 %, while in *S. lycopersicum* cv. ‘Ailsa Craig’ was only 26 % (Gharbi *et al.* 2017). The concentration of proline in the cv. ‘Ailsa Craig’ was 2.5-fold higher in the leaves than that of the wild relative, but concentrations in the roots were similar (Gharbi *et al.* 2017). The concentration of proline in the leaves has been negatively correlated with Na^+^ accumulation (Almeida *et al.* 2014).

### Antioxidant activity

Increases in salt concentration in plant tissue lead to an increase in the production of ROS (reviewed in Abogadallah 2010), mainly in organelles, plasma membrane and apoplast (You and Chan 2015). Reactive oxygen species have important functions in sensory and signalling networks during both normal plant developmental and stress responses (Noctor *et al.* 2014). At high concentrations, ROS become toxic and lead to oxidative stress which can interfere with the proper functioning of organelles such as chloroplasts and mitochondria, and induce damage to nucleic acids, proteins and enzymes, decrease membrane integrity, alter redox homeostasis thereby inducing photoinhibition and induce the activation of programmed cell death (Scandalios 1993). To minimize damage from ROS, plants have developed enzymatic and non-enzymatic antioxidant systems. An effective antioxidant system has been found to be responsible for enhanced salt tolerance in some wild tomato species. For example, increased activities of superoxide dismutase (SOD), catalase (CAT) and ascorbate peroxidase (APX), as well as increased levels of the reduced form of ascorbate and glutathione, were correlated with decreased lipid peroxidation in the salt-tolerant *S. pennellii* (accession Atico) when compared to tomato cv. ‘M82’ under salt stress (Shalata *et al.* 2001; Mittova *et al.* 2004). A separate study of 50 introgression lines (ILs) and their parental lines, *S. pennellii* (accession LA716) and tomato cv. ‘M82’, did not reveal a clear positive relationship between the induction of antioxidant enzymes under salt stress (150 mM NaCl, about 15 dS m^−1^) and plant growth (Frary *et al.* 2010). Most ILs had lower plant height, leaf number and leaf dry weight, but increased stem diameter and root growth under salt stress, which was a characteristic response of the salt-tolerant parent (i.e. *S. pennellii*; Frary *et al.* 2010).

## Conclusion

Improving salt tolerance in tomato can lead to substantial, positive economic and ecological impacts on horticultural production. The salt tolerance mechanism of the domesticated tomato (glycophytic-like response) is efficient under mild salt stress, but fails at higher salinity levels or during longer exposures to salt stress (Albaladejo *et al.* 2017; Meza *et al.* 2020). Tomato wild relatives, which often exhibit halophytic-like behaviours, show higher growth rates at high salt concentrations (Bolarin *et al.* 1991; Albaladejo *et al.* 2017; Pailles *et al.* 2020); however, they show low capacity for biomass accumulation and yield (Foolad 1996; Tal 1997). Under commercial production, where C assimilation and biomass accumulation is key for high yields, salt tolerance should aim to maintain favourable plant water status by a sustained capacity for uptake of water and nutrients. One approach to sustain C assimilation could be through the improvement of root functional traits per unit root area. For instance, the increase in *Lp*_r_ and the Na^+^ exclusion mechanisms can allow the influx of low-osmotic-potential water to the roots and maintain a better leaf water status (Ashraf and Shahbaz 2003; Moshelion *et al.*, 2017; Ashraf *et al.*, 2018). Although breeding for salt tolerance in tomato has proven challenging, this review demonstrates that tomato wild relatives possess potentially beneficial mechanisms for coping with salinity, and the identification of leaf and root traits and future integration with breeding efforts and agronomic techniques (e.g. use of rootstocks) could help overcome the transfer of undesirable traits to elite cultivars.

## Data Availability

All data and coding used in this study are available at the following link: https://osf.io/3hcmz/?view_only=9dabdc47126a4761b8df676e4e5952a9.

## Notes

**Form & Function. Chief Editor:** Kate McCulloh
